# Malignant peripheral nerve sheath tumor mimicking carotid body tumor, a rare case report and review of literature

**DOI:** 10.1016/j.ijscr.2024.110406

**Published:** 2024-10-02

**Authors:** Pezhman Kharazm, Soheil Noruzi, Alireza Aghili, Ali Zarbakhsh, Ramin Azarhoosh, Fereshteh Maghsudloo

**Affiliations:** a Assistant Professor of Vascular Surgery, Clinical Research Development Center, 5 Azar Hospital, Golestan University of Medical Sciences, Gorgan, Iran; b Vascular Surgeon, Clinical Research Development Center, 5 Azar Hospital, Golestan University of Medical Sciences, Gorgan, Iran; c Resident of General Surgery, Clinical Research Development Center, 5 Azar Hospital, Golestan University of Medical Sciences, Gorgan, Iran; d Assistant Professor of Pathology, Clinical Research Development Center, 5 Azar Hospital, Golestan University of Medical Sciences, Gorgan, Iran; e Assistant Professor of Radiology, Clinical Research Development Center, 5 Azar Hospital, Golestan University of Medical Sciences, Gorgan, Iran

**Keywords:** Neck mass, Carotid body tumor, Malignant peripheral nerve sheath tumor, Case report

## Abstract

**Introduction and importance:**

Malignant Peripheral Nerve Sheath Tumor (MPNST) is a rare type of soft tissue sarcoma. It is an aggressive tumor with high rates of local recurrence and distant metastasis. MPNST rarely occurs in the neck. We present a case of cervical MPNST manifesting as Carotid Body Tumor (CBT).

**Case presentation:**

A 67-year-old man presented with a neck mass. The mass was rapidly enlarging and imaging studies favored CBT. A previous attempt at surgical resection failed, and the compressive symptoms were progressive during recent weeks. After multidisciplinary discussion, the tumor was resected and pathological evaluation confirmed the diagnosis of MPNST. Post-operative metastatic work-up showed lung metastasis, and the patient died approximately one year after surgery.

**Clinical discussion:**

Cervical MPNST is rare, and surgery is the mainstay of its treatment. Pre-operative tissue diagnosis is recommended when possible, and immunohistochemical staining is necessary for prompt diagnosis. Adjuvant therapy may be helpful in metastatic cases or incomplete resection. Nevertheless, local recurrence and distant metastasis especially to the lungs are common, as in our case.

**Conclusion:**

MPNST is one of the potential causes of cervical masses and considering its invasive behavior, surgical resection is recommended as soon as the diagnosis is made.

## Highlights

• Malignant Peripheral Nerve Sheath Tumor (MPNST) is a rare type of soft tissue sarcoma.
• Few cases of cervical MPNST have been reported in the literature.
• MPNST has aggressive behavior and a severe propensity to invade adjacent structures, especially in the neck.
• MPNST of the vagus nerve can splay the angle between internal and external carotid arteries, resembling Carotid Body Tumor
• Margin-negative surgical resection is the mainstay of treatment for MPNST.

## 1 Introduction

Malignant Peripheral Nerve Sheath Tumor (MPNST) is a rare type of soft tissue sarcoma (STS) originating from peripheral nerve or nerve sheath cells. These tumors account for 5 % to 10 % of all soft tissue sarcomas [1,2]. Most MPNSTs are associated with neurofibromatosis type 1 (*NF1*), whereas others are post-radiation or sporadic tumors. The aggressive behavior of MPNST is the cause of the poor survival rate of the patients involved [3]. Few cases of cervical MPNST have been reported in the literature, and most of these tumors are located in the trunk and extremities [4]. Surgery is the mainstay of treatment for MPNST. Radiation therapy and systemic chemotherapy have minimal effects on overall survival [5]. In this study, we present a case of cervical mass operated with the presumed diagnosis of Carotid Body Tumor (CBT), but pathologic evaluation was in favor of MPNST.

This case report is reported in line with the SCARE Criteria [6].

## 2 Case presentation

**Fig. 1:**
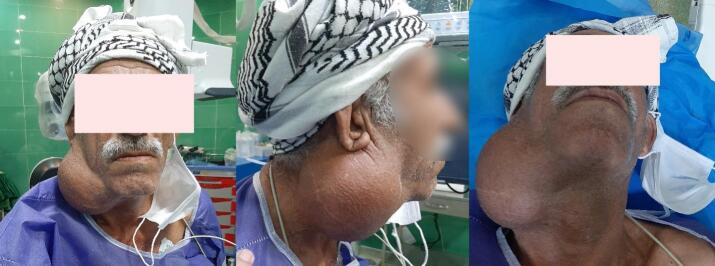
The large mass on the right side of the neck on different views. The scar from the previous surgery is visible.

A 67-year-old man presented with a neck mass. He had noticed the mass in his neck two years ago. It was rapidly enlarging and 6 months after the mass appearance, the patient had been operated on in another center with the presumed diagnosis of carotid body tumor, but the mass was unresectable, and considering the diagnosis of CBT, no attempt to take tissue biopsy was made. He was referred to other centers after this unsuccessful operation, but due to the advancement of the tumor and adherence to adjacent vital structures, it was considered inoperable. The patient was referred to our center and evaluated at a multidisciplinary consultative conference (Tumor Board). On physical examination, the patient had significant stridor during inspiration, in addition to hoarsness during the recent weeks. Mild dysphagia without complaint of aspiration was also observed. On inspection, a mass measuring 10 × 10 cm was visible on the right side of the neck. It had filled the angle of the mandible, extending anteriorly to approximately 3 cm from the chin (Fig. 1). The mass was hard and mobile in the lateral direction but fixed in the upward and downward directions. The skin was normal and pliable over the mass. The other parts of the examination were unremarkable.

**Fig. 2:**
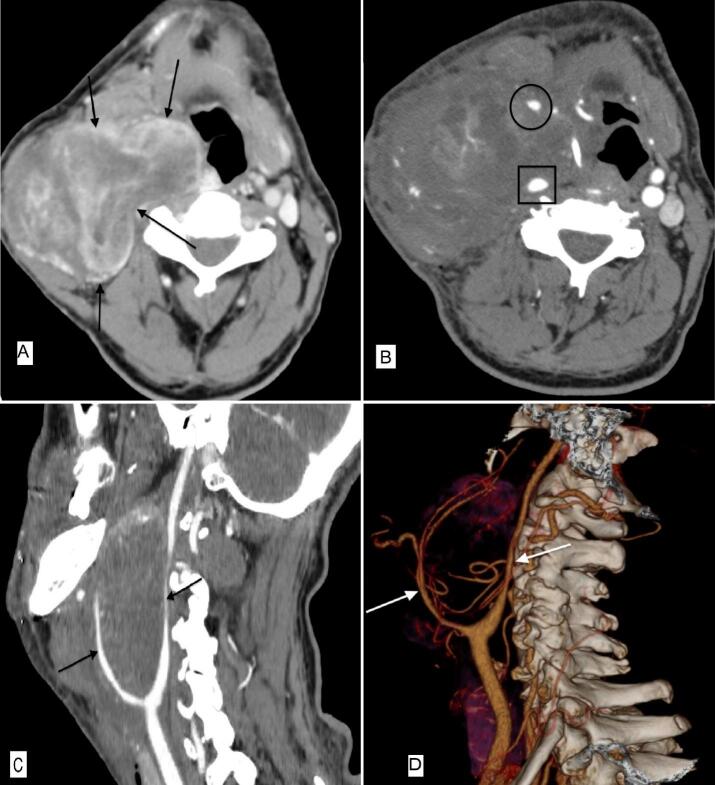
A. Axial contrast-enhanced CT images of the neck demonstrate heterogeneous enhancing well-demarcated mass on the right side with deep extension to the parapharyngeal space and significant compression effect on adjacent structures (arrow). B. Axial CTA of the patient confirms the mass hypervascularity, multiple internal arterial branches, and also splaying of the internal (rectangle) and external (circle) arteries. Sagittal (C) and volume-rendering (D) CTA reconstruction again demonstrate splaying of the internal and external carotid arteries (arrows).

On CT Angiography, a 9 × 10 × 9 cm mass was present on the right side of the neck. The mass splayed the angle between the internal and external carotid arteries, compressed the adjacent structures, and deviated the trachea to the left side (Fig. 2).

After a comprehensive multidisciplinary consultation, the patient was scheduled for another chance for resection by the vascular surgery team, considering his stridor and impending airway obstruction with the presumed diagnosis of the Carotid Body Tumor (CBT).

In the operating room, pre-operative direct laryngoscopy was performed. In addition to tracheal deviation, the right vocal cord was fixed in paramedian position without any movement.

Under general anesthesia and after skin preparation and draping, a 12-centimeter-long incision was made anterior to the sternocleidomastoid muscle. The mass was located directly under the platysma muscle, and had an intact capsule. There were some fibrotic adhesions to the tumor capsule because of previous surgery that were released. Considering the presumed diagnosis of the CBT, the common carotid artery was explored and controlled with vascular tape to perform peri-adventitial dissection of the tumor. However, further exploration revealed that, although the tumor was located between the internal and external carotid arteries, the capsule was separated from the arterial wall.

**Fig. 3:**
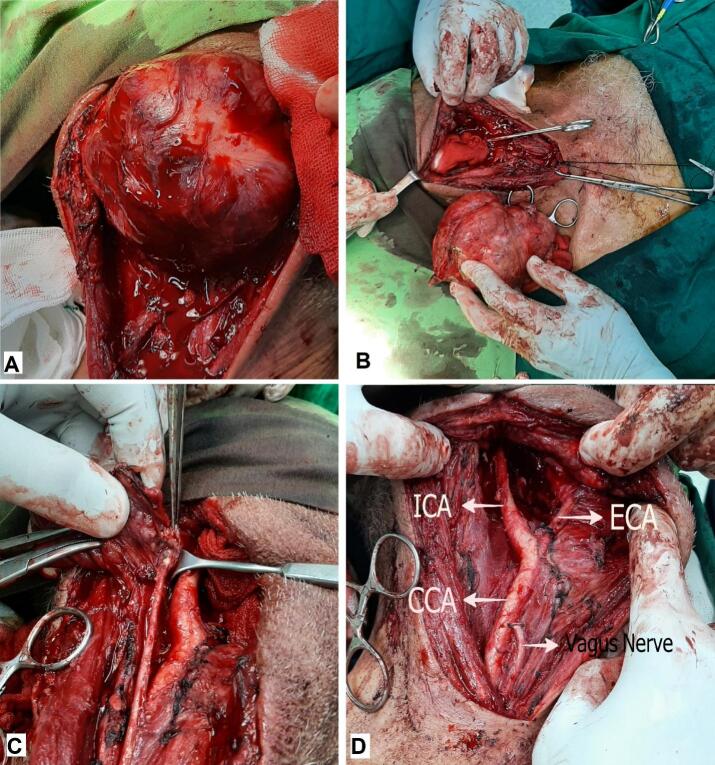
A. The mass was encapsulated and separated from arterial adventitia. B. The mass was resected en-bloc with an intact capsule. C. The vagus nerve was invaded by the second tumor and considering the pre-operative nerve palsy, it was resected segmentally with the tumor. D. Operation field at the end of surgery.

Meticulous extracapsular dissection separated the mass from the carotid arteries. The internal jugular vein had dense adhesions to the tumor and then, it was dissected en-bloc with the tumor after suture ligation of the vein near its confluence with the right subclavian vein. After dissection of the distal end of the jugular vein, another small mass was encountered which was separated from the original mass encasing the vagus nerve. Isolation of the nerve from the mass was impossible, and considering pre-operative laryngoscopy, both of the masses were resected en-bloc with segments of the involved nerve and vein (Fig. 3).

**Fig. 4:**
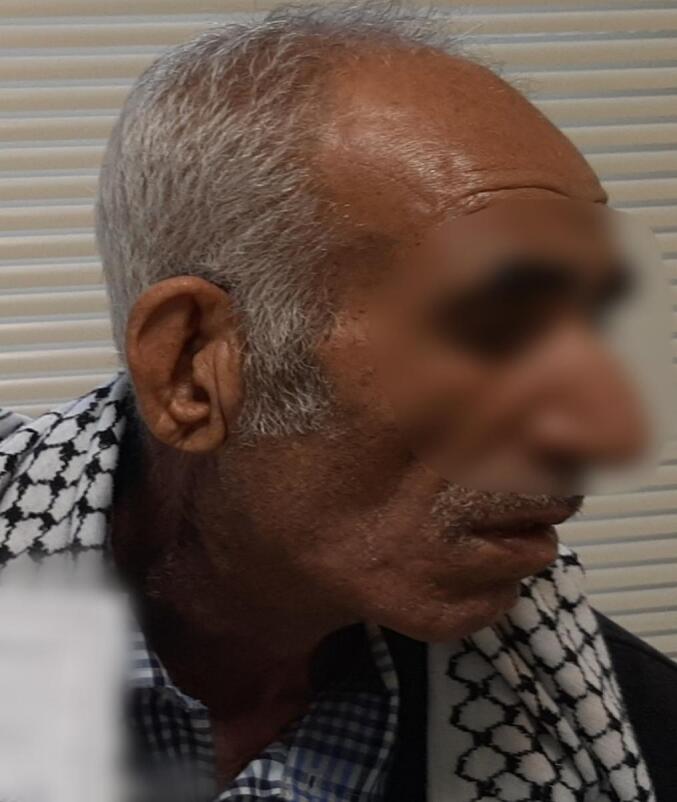
The patient's symptoms were significantly reduced after surgery.

After hemostasis, the incision was closed using a closed suction drain. The post-operative laryngoscopic evaluation was the same as the pre-operative evaluation, and after confirmation of adequate respiratory function, the patient was extubated and transferred to the SICU for post-operative monitoring. Stridor and dysphagia significantly reduced, but hoarsness persisted. The drain was removed 48 h later at the time of patient's discharge. On early post-operative days, the patient had cough attacks following liquid meals or even swallowing of saliva; however, this problem gradually resolved spontaneously (Fig. 4).

**Fig. 5:**
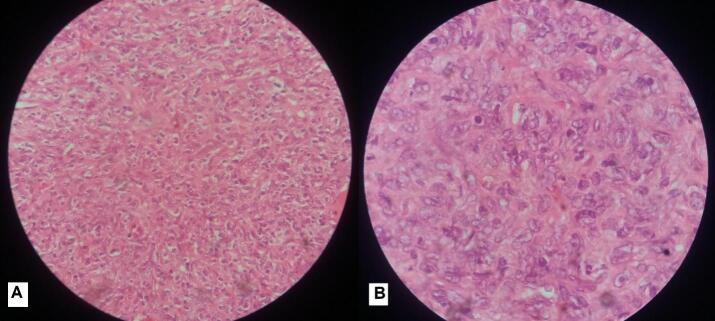
A. Sheets of large polygonal tumor cells with interspersed fibrocollagenous septa. These cells showed moderate pleomorphism, abundant glassy to eosinophilic cytoplasm with prominent eosinophilic macro-nucleoli, and increased mitoses. B. The neoplastic cells were ovoid to polygonal, with abundant eosinophilic cytoplasm. The cells had moderate to severe atypia, with irregular nuclei with clumped and/or vesicular chromatin, and conspicuous nucleoli.

Pathological evaluation of the mass, including immunohistochemical analysis, revealed the diagnosis of a malignant peripheral nerve sheath tumor (Fig. 5).

After pathological confirmation of the diagnosis, the patient was referred for metastatic workup and adjuvant therapy. Chest CT-scan revealed multiple lung metastases. Therefore, the patient was scheduled to receive adjuvant chemotherapy and cervical radiotherapy for local control.

Unfortunately, the disease was not controlled, and 11 months later, the patient died because of local tumor recurrence and metastasis.

## 3 Discussion

Malignant peripheral nerve sheath tumors (MPNST) are rare, with an estimated incidence of 0.001 % [7]. Because of the rarity of this tumor, there are only a few reports, and little information is available on MPNST occurring in the head and neck area [8]. This rarity in addition to the typical manifestation of CBT, made the pre-operative diagnosis of MPNST unexpected in our patient.

The peak age of onset for MPNST is 30–40 years, and more than half of cases arise in patients with neurofibromatosis 1(NF1) [7]. Our patient was older, and the post-operative work-up was negative for neurofibromatosis.

MPNST tumors are most commonly located in the extremities (45 %), trunk (34 %), and head/neck (19 %) [1]. The rates of local recurrence and distant metastasis are very high, and distant metastasis mainly occurs in the lungs [7]. Post-operative work-up in our case confirmed lung metastasis and despite complete removal of the tumor and adjuvant treatment, local recurrence and metastasis spread resulted in the patient's death.

The clinical features of MPNSTs include rapid growth of the mass, accompanied by pain, and compressive symptoms [3]. This patient had a rapidly enlarging mass with dysphagia, hoarseness, and respiratory stridor as the compressive signs and symptoms.

Although computed tomography (CT) scan, magnetic resonance imaging (MRI), and positron emission tomography (PET) can diagnose suspicious MPNSTs with significant accuracy, histopathology and immunohistochemistry are necessary to confirm the diagnosis. Therefore, pre-operative tissue diagnosis using core needle aspiration is recommended in suspected cases [5]. In our case, imaging findings favored a Carotid Body Tumor (CBT). Considering this diagnosis and the severe compressive symptoms, pre-operative tissue diagnosis was ignored, and the patient was directly scheduled for definite surgery. Pathological evaluation and immunohistochemical analysis were consistent with the diagnosis of MPNST.

The mainstay of treatment for MPNST is surgical resection, with the goal of achieving complete removal with negative margins such as all soft tissue sarcomas. Chemotherapy is recommended for patients with metastatic or unresectable tumors. Chemotherapy in the setting of neoadjuvant or adjuvant therapy does not affect survival or local recurrence rates and is not recommended [9]. Radiation therapy is an important adjunct to surgery for improving local control and may be administered in neoadjuvant or adjuvant settings [1].

Considering the rarity of MPNSTs in the neck, there are a limited number of studies in this regard. Table 1 briefly shows available studies and their findings regarding cervical MPNST [7,[10], [11], [12], [13], [14], [15], [16]].

**Table 1:** Characteristics and findings of available studies regarding cervical MPNST.

Author	Journal	Year	Number of cases	5-year-survival	NF prevalence	Metastasis	Mean age	Men/women ratio
K. Boumaza et al.	European Annals of Otorhinolaryngology, Head and Neck diseases	2019	73	50 %	30 %	N\A	44	0.825
Yan, P. H. et al.	Journal of Neuro-Oncology	2019	689	55.9 %	N\A	13.2 %	45.8	1.026
van Noesel, M. M. et al.	Pediatric Blood & Cancer	2019	51	52.9 %	51 %	11.7 %	13.7	0.96
Watson, K. L. et al.	Journal of neurosurgery	2017	289	52 %	51 %	10.3 %	37	1.15
Miao, R. Y. et al.	Elsevier B.V. Radiotherapy and Oncology	2019	280	51.9 %	27.8 %	10.4 %	41	0.97
Mowery, A. et al.	Elsevier oral oncology	2019	2858	52 %	N\A	3.7 %	47	1.19
Martin, E. et al.	European Journal of Cancer	2020	784	N\A	26.8 %	11.5 %	49	1.15
Hwang, I. K. et al	Cancer Res Treat	2017	95	52 %	34.7 %	9.4 %	40.4	1.11

Post-operative work-up in our patient confirmed the presence of lung metastases. The patient was scheduled for chemotherapy and radiotherapy during which time he experienced local recurrence and metastasis spread and died approximately one year after surgery.

## 4 Conclusion

In patients with a rapidly enlarging neck mass, MPNST is an important differential diagnosis, even in the absence of NF1. Multidisciplinary discussions are crucial in defining the best treatment plan. Surgery is the most effective treatment for these patients, and the best prognoses belong to patients whose operations are performed before metastatic spread, especially if the patient has symptoms of compression of the vital organs. Considering the behavior of the MPNST and the invasion of nearby organs, surgery may be very challenging in these patients, and it is recommended that it be performed by an experienced surgeon familiar with any potential complications.

## Consent

Consent was obtained for the publication of this article and images from the patient's next of kin because of the patient's death.

## Ethical approval

This study was approved by the Golestan University of Medical Sciences Research Ethics Committee with the following ethics code: https://ethics.research.ac.ir/IR.GOUMS.REC.1403.192. Date of approval: 2024-08-20.

## Funding

There is no source of funding for this article.

## Author contribution

Pezhman Kharazm, Vascular Surgeon and the patient's surgeon in charge.

Soheil Noruzi, Vascular Surgeon, member of surgery team.

Alireza aghili, Resident of General Surgery, literature review, article writing.

Ali Zarbakhsh, Resident of General Surgery, literature review, article writing.

Ramin Azarhoosh, Pathologist, pathology report and photo provider.

Fereshteh Maghsudloo, Radiologist, CTA report and photo provider.

## Guarantor

Dr. Pezhman Kharazm.

## Conflict of interest statement

No conflict of interest is present between authors.
